# eEF-2K Deficiency Boosts the Virus-Specific Effector CD8^+^ T Cell Responses During Viral Infection

**DOI:** 10.3390/v17010026

**Published:** 2024-12-28

**Authors:** Liqing Wang, Benny Shone Song, Rayansh Poojary, Xiaofang Xiong, Xingcong Ren, Jin-Ming Yang, Jianxun Song

**Affiliations:** 1 Department of Microbial Pathogenesis and Immunology, Texas A&M University Health Science Center, Bryan, TX 77807, USArayansh.poojary.888@k12.friscoisd.org (R.P.); xxiong@tamu.edu (X.X.); 2 Department of Toxicology and Cancer Biology, University of Kentucky College of Medicine, Lexington, KY 40536, USA

**Keywords:** eEF-2K, effector CD8^+^ T cells, vaccinia virus (VACV), T cell immunity, TRAF3, viral infection

## Abstract

In this study, we revealed a critical role of eukaryotic elongation factor-2 kinase (eEF-2K), a negative regulator of protein synthesis, in regulating T cells during vaccinia virus (VACV) infection. We found that eEF-2K-deficient (eEF-2K⁻/⁻) mice exhibited a significantly higher proportion of VACV-specific effector CD8^+^ T cells without compromising the development of VACV-specific memory CD8^+^ T cells. RNA sequencing demonstrated that eEF-2K⁻/⁻ VACV-specific effector CD8^+^ T cells had enhanced functionality, which improves their capacity to combat viral infection during the effector phase. Moreover, we identified tumor necrosis factor receptor-associated factor 3 (TRAF3) as a critical mediator of the stronger antiviral response observed in eEF-2K⁻/⁻ effector CD8^+^ T cells. These findings suggest that targeting eEF-2K may provide a novel strategy to augmenting effector CD8^+^ T cell responses against viral infections.

## 1. Introduction

T cells are essential to immune defense against viral infections. Upon viral invasion, antigen-presenting cells present viral antigens to T cells, initiating a robust immune response. Virus-specific CD8^+^ T cells play a critical role by directly targeting infected cells and secreting cytotoxic molecules including IFN-γ, TNF-α, and granzyme B to eliminate the pathogen [1,2]. The host immune response progresses through distinct phases: expansion of T cells during the effector stage, contraction to regulate immune homeostasis, and the formation of long-lived memory T cells [3]. The effectiveness of the response is affected by various factors such as transcriptional regulators, kinases, and co-stimulatory molecules, which collectively shape the antiviral T cell response [4,5,6,7]. Despite substantial advancement, how to strengthen antiviral immunity remains a major challenge. Most of the current efforts focus on bolstering effector T cell responses and optimizing memory T cell formation, particularly in the context of vaccination.

Eukaryotic Elongation Factor-2 Kinase (eEF-2K) is a critical negative regulator of protein synthesis that inhibits translation elongation under cellular stressful conditions [8]. Although eEF-2K has been studied extensively in cancer [9,10,11,12,13,14,15] and some other diseases [16,17,18,19,20], its role in immune responses, particularly in T cells, remains underexplored. In cancer, eEF-2K has been implicated in various cellular processes, including tumor cell proliferation, migration, and metastasis. Recently, emerging evidence suggested that eEF-2K can modulate immune responses. Previous studies from our laboratory demonstrated that eEF-2K plays a critical role in CD8^+^ T cell- and NK cell-mediated antitumor immunity [21,22], and its absence in CD4^+^ T cells promotes an inflammatory response characterized by enhanced Th17 polarization [23]. Despite these findings, the precise mechanisms by which eEF-2K regulates T cell responses during viral infections remain poorly understood. In this study, we investigated the role of eEF-2K in CD8^+^ T cell responses during vaccinia virus (VACV) infection. We demonstrated that the EF-2K-deficient (eEF-2K⁻/⁻) mice exhibited an increased proportion of VACV-specific effector CD8^+^ T cells without impairing the formation of memory CD8^+^ T cells. RNA sequencing showed that eEF-2K⁻/⁻ VACV-specific effector CD8^+^ T cells had enhanced functionality, rendering them more effective during the effector phase of viral infection. Additionally, we identified TRAF3 as a likely mediator of the boosted antiviral response observed in eEF-2K⁻/⁻ VACV-specific effector CD8^+^ T cells. These results suggest that targeting eEF-2K may provide a novel strategy to enhance effector CD8^+^ T cell responses during viral infections.

## 2. Materials and Methods

### 2.1. Animal Experiments

C57BL/6 (B6) mice were obtained from The Jackson Laboratory (Bar Harbor, ME, USA). eEF-2K-deficient (eEF-2K⁻/⁻) mice on a B6 background were bred and housed in the Texas A&M University Laboratory Animal Resources and Research Facility. All experiments used mice aged 8–12 weeks. Procedures involving animals adhered to protocols approved by the Institutional Animal Care and Use Committee (IACUC #2021-0123), Texas A&M University.

### 2.2. Virus Preparation and Titration

Vaccinia virus Western Reserve strain (VACV-WR) stocks were propagated in HeLa cells. When HeLa cells reached ~95% confluence, they were infected with VACV at a multiplicity of infection (MOI) of 2 PFU/cell. Two days post-infection, cells and viral particles were harvested via repeated freeze–thaw cycles. Viral stocks were titrated by plaque assays using Vero C1008 cells. Briefly, confluent Vero cells were incubated with serial dilutions of the viral stock in 6-well plates for two days, and plaques were visualized and quantified using crystal violet staining. Detailed methods for virus propagation and titration were based on a previously published protocol [24]. Viral stocks were stored at −80 °C for long-term use.

### 2.3. Viral Infection

VACV stocks were diluted in sterile PBS and administered to mice via intraperitoneal injection at a dose of 2 × 10⁶ PFU/mouse, as previously described. The frequency and phenotype of VACV-specific CD8⁺ T cells were monitored over a 35-day period post-infection.

### 2.4. Tissue Processing

Spleens and pooled lymph nodes from each mouse were harvested and mechanically dissociated into single-cell suspensions using a 40 μm cell strainer. Red blood cells were lysed using Pharm Lyse™ Lysing Buffer (BD Biosciences, San Diego, CA, USA). Total viable cells were counted using a Bio-Rad TC20™ automated cell counter.

### 2.5. Flow Cytometry and Tetramer Staining

Single-cell suspensions from spleen and lymph nodes were stained for surface markers in staining buffer (BioLegend, San Diego, CA, USA) on ice for 15 min in the dark [25]. VACV-specific CD8⁺ T cells were identified using an MHC class I B8R (TSYKFESV) tetramer synthesized by the NIH Tetramer Core. Tetramer staining was performed at room temperature for 30 min in the dark. Flow cytometry was conducted on a BD Fortessa X-20 cytometer at the Texas A&M University College of Medicine Cytometry and Flow (COM-CAF) Core Facility. Antibody details are listed in Table 1, and data analysis was performed using FlowJo software.

### 2.6. Sorting of VACV-Specific CD8⁺ T Cells

VACV-specific CD8⁺ T cells were sorted from spleens and lymph nodes 14 days post-infection using a BD FACS Aria II cell sorter in the Texas A&M University College of Medicine Analytical Cytometry Core (SMACC). Sorted cells were used for RNA sequencing.

### 2.7. RNA Sequencing and Data Analysis

Sorted VACV-specific CD8⁺ T cells from wild-type (WT) and eEF-2K⁻/⁻ mice (*n* = 3 biological replicates per group) were subjected to bulk RNA sequencing. RNA integrity was assessed using an Agilent 2100 Bioanalyzer. Library preparation and sequencing were performed by Novogene (Sacramento, CA, USA). Differential gene expression (DEG) and gene ontology (GO) analyses were conducted using R Studio.

### 2.8. Intracellular Cytokine and Protein Staining

Following surface marker and VACV tetramer staining (as described in Section 2.5), cells were fixed with fixation buffer (BioLegend, San Diego, CA, USA) at room temperature for 20 min in the dark. Permeabilization was performed twice using permeabilization buffer (BioLegend, San Diego, CA, USA) at 2000× *g* for 10 min each. Intracellular cytokine and protein staining (e.g., IFN-γ, IL-2, BCL-2) was performed in permeabilization buffer, with staining times optimized for each target molecule.

### 2.9. RNA Extraction and Quantitative PCR (qPCR)

Total RNA was extracted from VACV-specific CD8⁺ T cell pellets using the RNeasy Mini Kit (Qiagen, Germantown, MD, USA) and treated with the TURBO DNA-free Kit (Ambion, Austin, TX, USA) to eliminate genomic DNA contamination. cDNA synthesis was performed using the High-Capacity cDNA Reverse Transcription Kit (Thermo Fisher, Waltham, MA, USA). qPCR was performed using the following primers:

- Traf3 Forward: GTGAACCTGCTGAAGGAGTGGA
- Traf3 Reverse: TTCGGAGCATCTCCTTCTGCCT
- Gapdh Forward: GTTGTCTCCTGCGACTTCA
- Gapdh Reverse: GGTGGTCCAGGGTTTCTTA

### 2.10. Illustration and Figures

Bar graphs were generated using GraphPad Prism software. Flow cytometry plots were visualized with FlowJo software. Figures for DEG and GO analyses were created in R Studio, and illustrations were designed using BioRender Online.

### 2.11. Statistical Analysis

Statistical analyses were performed using Student’s *t*-test, with *p*-values < 0.05 considered statistically significant.

## 3. Results

### 3.1. eEF-2K⁻/⁻ Mice Show Enhanced Response to VACV Infection During the Effector Stage

**Figure 1:**
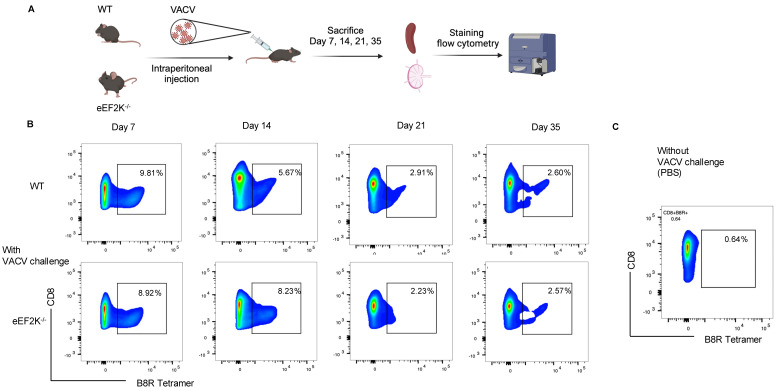
Development of VACV-specific CD8⁺ T cells. The frequency of VACV-specific CD8⁺ T cells was tracked in WT and eEF-2K⁻/⁻ mice for 35 days post-VACV challenge (2 × 10⁶ PFU/mouse). Spleens and lymph nodes (LNs) were harvested, processed, and stained with CD8 antibodies and B8R tetramer. (**A**) Schematic representation of the experimental timeline. (**B**) Time-course analysis of VACV-specific CD8⁺ T cell frequencies on days 7, 14, 21, and 35, as determined by flow cytometry. (**C**) VACV-uninfected mice served as controls for baseline comparison. Representative data are shown from three independent experiments (*n* = 5 mice/group).

**Figure 2:**
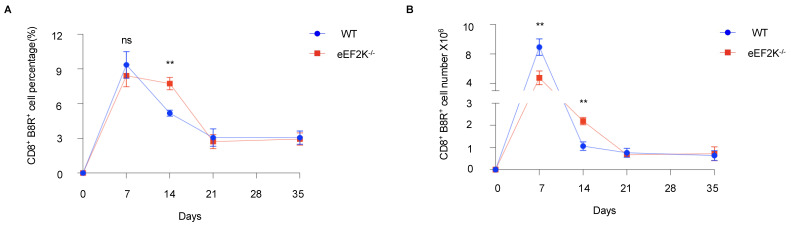
Sustained numbers and frequencies of VACV-specific effector CD8⁺ T cells in eEF-2K⁻/⁻ mice. Changes in the numbers and frequencies of VACV-specific CD8⁺ T cells were analyzed in WT and eEF-2K⁻/⁻ mice over 35 days post-infection. (**A**) Total numbers of CD8⁺B8R⁺ T cells in spleens and LNs at days 7, 14, 21, and 35 post-infection (*n* = 5 mice/group). (**B**) Frequencies of CD8⁺B8R⁺ T cells 35 days post-infection. **, *p* < 0.01; ns, not significant. Data are representative of three independent experiments.

Our previous studies demonstrated the higher proliferation of eEF-2K⁻/⁻ CD8⁺ T cells than that of wild-type (WT) cells upon activation [1]. To assess the role of eEF-2K in CD8⁺ T cell responses to viral infection, we infected WT and eEF-2K⁻/⁻ mice with 2 million PFU of VACV, and then analyzed CD8⁺ B8R⁺ T cells for over 35 days (Figure 1A). Both WT and eEF-2K⁻/⁻ mice generated robust responses, as evidenced by increased CD8⁺ B8R⁺ T cells as compared to the PBS controls (Figure 1B,C). At day 7 post-infection, similar percentages of CD8⁺ B8R⁺ T cells (~9%) were observed in both WT and eEF-2K⁻/⁻ mice (Figure 1B and Figure 2A), although total CD8⁺ B8R⁺ cell numbers were lower in eEF-2K⁻/⁻ mice due to smaller spleens and lymph nodes. By day 14, eEF-2K⁻/⁻ mice exhibited a significantly higher proportion of CD8⁺ B8R⁺ cells (Figure 1B and Figure 2A), with improved survival of these cells compared to the WT counterparts (Figure 2B). These results indicate that eEF-2K deficiency improves the survival and antiviral potency of CD8⁺ T cells during the effector stage.

### 3.2. eEF-2K Deficiency Does Not Influence VACV-Specific Memory T Cell Formation

**Figure 3:**
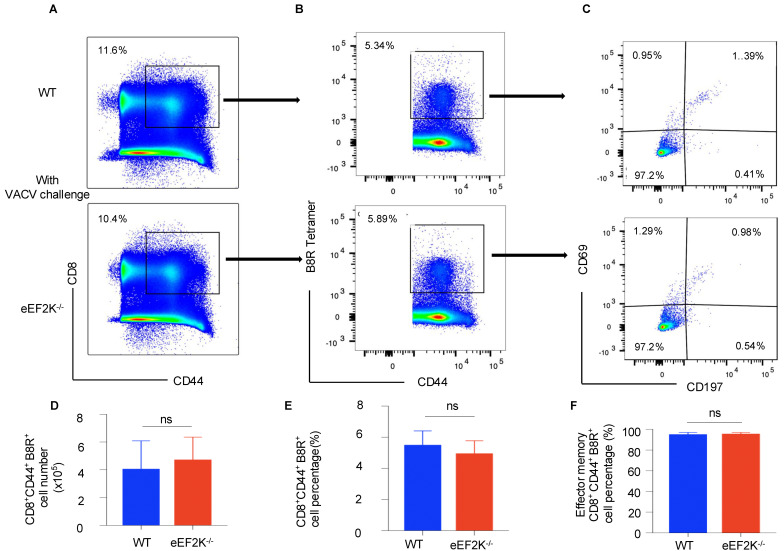
eEF-2K deficiency does not impair memory CD8⁺ T cell formation during VACV infection. VACV-specific memory CD8⁺ T cells were analyzed 35 days post-infection in WT and eEF-2K⁻/⁻ mice. (**A**) Memory CD8⁺ T cells (CD8⁺CD44⁺). (**B**) VACV-specific memory CD8⁺ T cells (CD8⁺CD44⁺B8R⁺), gated on memory CD8⁺ T cells (CD8⁺CD44⁺). (**C**) Subsets of memory T cells: tissue-resident memory (CD44⁺CD69⁺CD197⁻), effector memory (CD44⁺CD69⁻CD197⁻), and central memory (CD44⁺CD69⁻CD197⁺). (**D**) Total numbers of VACV-specific memory CD8⁺ T cells (CD8⁺CD44⁺B8R⁺). (**E**) Frequencies of VACV-specific memory CD8⁺ T cells (CD8⁺CD44⁺B8R⁺). (**F**) Frequencies of effector memory T cells (CD44⁺CD69⁻CD197^−^).

To evaluate the role of eEF-2K in memory T cell formation, we analyzed WT and eEF-2K⁻/⁻ mice at day 35 post-infection. The frequencies and numbers of VACV-specific CD8⁺ T cells were comparable between the two groups (Figure 2A,B). Flow cytometric analysis revealed no differences in the generation of virus-specific CD8⁺ B8R⁺ CD44⁺ memory T cells (Figure 3A,B), and both groups displayed similar effector memory T cell populations (CD8⁺ B8R⁺ CD44⁺ CD69⁻ CD197⁻) (Figure 3C,F). These observations suggest that eEF-2K is not required for formation or maintenance of virus-specific memory T cells during VACV infection.

### 3.3. Involvement of Transcriptional Alteration in the Augmented Response of VACV-Specific eEF-2K⁻/⁻ Effector CD8⁺ T Cells to Viral Infection

**Figure 4:**
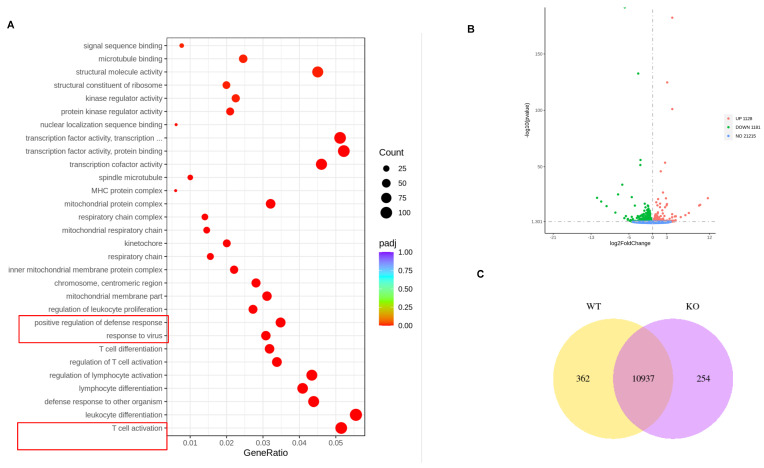
Transcriptomic analysis of eEF-2K⁻/⁻ VACV-specific effector CD8⁺ T cells. VACV-specific effector CD8⁺ T cells were sorted from WT and eEF-2K⁻/⁻ mice 14 days post-infection for bulk RNA sequencing. Bioinformatic analysis was performed to explore differential gene expression and pathway enrichment. (**A**) Gene ontology (GO) enrichment analysis highlighting biological processes related to viral response. (**B**) Volcano plot showing differentially expressed genes between WT and eEF-2K⁻/⁻ CD8⁺ T cells. (**C**) Venn diagram illustrating overlapping genes involved in antiviral responses.

As we observed the enhanced responses of VACV-specific eEF-2K⁻/⁻ effector CD8⁺ T cells to viral infection (Figure 2), we performed RNA-sequencing of sorted VACV-specific CD8⁺ T cells and found a significant enrichment of pathways associated with antiviral defense, mitochondrial function, and respiratory chain activity in eEF-2K⁻/⁻ CD8⁺ T cells (Figure 4A). Alterations in genes involved in T cell activation and defense response was observed in eEF-2K⁻/⁻ CD8⁺ T cells, with 1,128 genes upregulated and 1181 downregulated compared to WT CD8⁺ T cells (Figure 4B,C). These results suggest that eEF-2K may regulate the transcriptional landscape of CD8⁺ T cells, orchestrating their antiviral activity and aligning with the superior responses observed in eEF-2K⁻/⁻ mice during VACV infection.

### 3.4. Functional Competence of VACV-Specific eEF-2K⁻/⁻ Effector CD8⁺ T Cells

**Figure 5:**
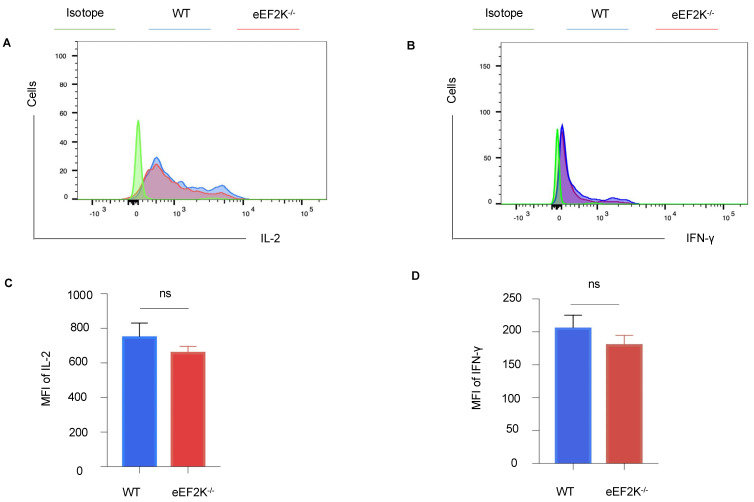
Functionality of eEF-2K⁻/⁻ VACV-specific effector CD8⁺ T cells. Cytokine production by VACV-specific effector CD8⁺ T cells was assessed in WT and eEF-2K⁻/⁻ mice. Spleens and LNs were harvested and stained with CD8, B8R tetramer, and intracellular IL-2 and IFN-γ antibodies. (**A**) Representative flow cytometry analysis of intracellular IL-2 secretion. (**B**) Representative flow cytometry analysis of intracellular IFN-γ secretion. (**C**) Mean fluorescence intensity (MFI) quantification of IL-2 production. (**D**) MFI quantification of IFN-γ production.

To evaluate functional capacity, we examined the cytokine secretion and anti-apoptotic marker expression in VACV-specific effector CD8⁺ T cells at day 14 post-infection. eEF-2K⁻/⁻ and WT CD8⁺ T cells secreted comparable levels of IL-2 and IFN-γ (Figure 5A–D), and expression of BCL-2, an anti-apoptotic marker, was similarly maintained in both groups (Supplementary Figure S1A,B). We have demonstrated that there is no significant difference in IFN-γ production between WT and eEF-2K⁻/⁻ CD8^+^ T cells, suggesting that the enhanced antiviral response observed in eEF-2K⁻/⁻ T cells is independent of IFN-γ. These findings indicate that eEF-2K⁻/⁻ effector CD8⁺ T cells retain functional competence and exhibit antiviral activity that is equivalent to WT cells.

### 3.5. TRAF3 Mediates the Enhanced Antiviral Response in eEF-2K⁻/⁻ Effector CD8⁺ T Cells

**Figure 6:**
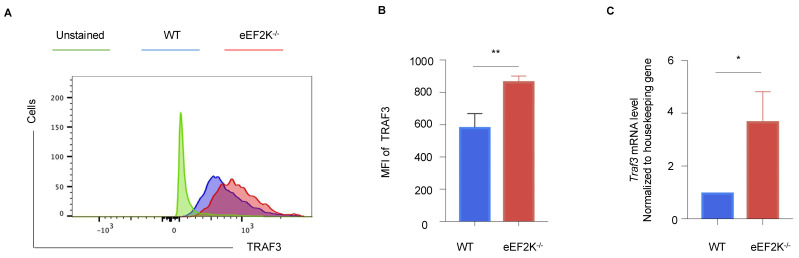
TRAF3 is involved in the eEF-2K-dependent regulation of antiviral immunity. Intracellular TRAF3 expression in VACV-specific effector CD8⁺ T cells was analyzed in WT and eEF-2K⁻/⁻ mice. (**A**) Representative flow cytometry plots of TRAF3 protein expression in CD8⁺B8R⁺ T cells. (**B**) MFI quantification of TRAF3 expression in CD8⁺B8R⁺ T cells. (**C**) TRAF3 mRNA levels in sorted CD8⁺B8R⁺ T cells from day 14 post-infection, normalized to housekeeping genes. *, *p* < 0.05; **, *p* < 0.01.

RNA-sequencing data identified TRAF3 as a critical regulator of pathways involved in defense response and antiviral activity (Supplementary Figure S2A). TRAF3 expression was significantly elevated at both mRNA and protein levels in VACV-specific eEF-2K⁻/⁻ effector CD8⁺ T cells (Figure 6A–C), which is consistent with its known role in promoting type I interferon responses during viral infections [26,27]. These results implicate TRAF3 in the enhanced antiviral response observed in eEF-2K⁻/⁻ CD8⁺ T cells and suggest that eEF-2K regulates antiviral immunity through a TRAF3-mediated pathway.

## 4. Discussion and Conclusions

This study demonstrates that eEF-2K acts as a critical regulator of virus-specific CD8^+^ T cell responses, particularly during the effector stage of vaccinia virus (VACV) infection. We show that eEF-2K deficiency enhances the percentage of VACV-specific CD8^+^ T cells during the effector stage, suggesting that eEF-2K negatively impacts the proliferation and survival of these immune cells (Figure 1 and Figure 2). This enhanced response aligns with our previous observations that eEF-2K⁻/⁻ CD8^+^ T cells undergo rapid proliferation upon in vitro activation [21]. Furthermore, transcriptomic analyses revealed that eEF-2K⁻/⁻ CD8^+^ T cells have an upregulation of the mitochondrial-related pathways, which are essential for T cell activation and effector function during viral infection (Figure 4A). This observation is consistent with our previous findings that enhanced mitochondrial functionality and elevated reactive oxygen species (ROS) production in eEF-2K⁻/⁻ CD4^+^ T cells contributed to stronger immune responses. Mitochondrial function plays a vital role in T cell activation and effector effect via amplifying signaling pathways essential for antiviral immunity. Thus, our results imply that eEF-2K can suppress the mitochondrial-mediated antiviral T cell responses [23].

eEF-2K functions as a negative regulator of protein synthesis by inhibiting translational elongation [8]. This mechanism may explain why its absence enables CD8^+^ T cells to rapidly proliferate and exhibit enhanced activation during VACV infection. However, our findings indicate that eEF-2K does not interfere with the formation of VACV-specific memory CD8^+^ T cells (Figure 3). Memory T cells typically exist in a resting state with lower metabolic and protein synthesis demands, which may render them less reliant on eEF-2K activity for their development and maintenance. This distinction highlights the selective role of eEF-2K in modulating T cell responses based on their activation status. We evaluated the functionality of VACV-specific eEF-2K⁻/⁻ CD8^+^ T cells by assessing their cytokine secretion and showed the comparable levels of IL-2 and IFN-γ produced by eEF-2K⁻/⁻ and WT CD8^+^ T cells (Figure 5), suggesting that absence of eEF-2K does not compromise the effector functions of CD8^+^ T cells. This study underscores the therapeutic potential of targeting eEF-2K to enhance effector T cell responses without impairing their antiviral capabilities. Moreover, the preservation of anti-apoptotic markers (e.g., BCL-2) in eEF-2K⁻/⁻ CD8^+^ T cells supports their functional integrity during the effector stage of VACV infection.

Also, our data implicate TRAF3, an adaptor protein involved in NF-κB pathway activation [28], as a key mediator of the enhanced antiviral response observed in eEF-2K⁻/⁻ CD8^+^ T cells (Figure 6). Elevated TRAF3 expression in eEF-2K⁻/⁻ CD8^+^ T cells suggests a potential link between eEF-2K and NF-κB-mediated immune regulation. TRAF3 has been previously reported to positively regulate immune responses, including type I interferon production, during influenza A virus infection [26]. Notably, we have also reported altered NF-κB activity in eEF-2K⁻/⁻ CD8^+^ T cells in the context of chimeric antigen receptor (CAR)–T cell therapy [21]. These findings suggest that TRAF3 plays a critical role in the eEF-2K-mediated regulation of CD8^+^ T cell responses during viral infection. As shown in Figure 5B,D, we have demonstrated that there is no significant difference in IFN-γ production between WT and eEF2K⁻/⁻ CD8^+^ T cells, suggesting that the enhanced antiviral response observed in eEF2K⁻/⁻ T cells is independent of IFN-γ. While we did not perform a direct IFN-γ assay in this study, our existing data indicate that the enhanced antiviral response in eEF2K⁻/⁻ CD8^+^ T cells is likely mediated through TRAF3-dependent mechanisms beyond IFN-γ production. Specifically, TRAF3 may influence antiviral immunity by regulating pathways such as IRF3 activation and ISG expression. Our current study identifies TRAF3 as a critical mediator of enhanced CD8^+^ T cell responses in eEF2K-deficient mice. However, the precise mechanisms by which eEF2K regulates TRAF3 remain to be elucidated. While our findings suggest a functional link between eEF2K deficiency and increased TRAF3 expression, the underlying molecular mechanisms remain unclear. Given eEF2K’s role as a kinase, one possibility is that it influences TRAF3 through post-translational modifications, such as phosphorylation or ubiquitination, which could alter TRAF3′s stability, localization, or interactions with signaling complexes. Alternatively, eEF2K might regulate TRAF3 at the transcriptional level by modulating signaling pathways or transcription factors that control TRAF3 expression, possibly through metabolic or stress-response pathways. Future studies will aim to investigate these mechanisms and uncover how eEF2K modulates TRAF3 expression and activity, providing deeper insights into the molecular crosstalk between eEF2K and TRAF3. Such investigations could refine eEF2K-targeted therapeutic strategies and identify additional targets for enhancing T cell immunity.

Our RNA-seq analysis highlighted mitochondrial signaling as a key pathway associated with the enhanced antiviral function of eEF-2K-deficient CD8^+^ T cells. Mitochondria play a central role in T cell metabolism, supporting both bioenergetic and biosynthetic demands during activation and differentiation. Notably, eEF-2K has been implicated in regulating cellular metabolism by modulating protein synthesis and responding to metabolic stress. In CD8^+^ T cells, these regulatory functions may influence mitochondrial dynamics, oxidative phosphorylation (OXPHOS), and glycolytic activity, all of which are critical for effective antiviral responses. eEF-2K deficiency may enhance mitochondrial function and metabolic flexibility, enabling CD8^+^ T cells to sustain robust effector functions. Increased mitochondrial signaling could drive the production of ATP and reactive oxygen species (ROS), supporting cytokine production, proliferation, and cytotoxic activity. Moreover, the metabolic shift facilitated by eEF-2K deficiency might promote memory precursor cell formation, as mitochondrial metabolism is essential for the development of long-lived memory T cells. Future studies should investigate how eEF-2K influences key metabolic pathways, such as the balance between glycolysis and OXPHOS, mitochondrial biogenesis, and ROS signaling, in CD8^+^ T cells during viral infections. Understanding these mechanisms could reveal novel strategies to optimize T cell metabolism for enhanced antiviral immunity.

While this study focuses primarily on the role of eEF-2K in CD8^+^ T cell responses during VACV infection, the impact of eEF-2K deficiency on CD4^+^ T cells remains an important area for exploration. CD4^+^ T cells are critical for coordinating immune responses, including providing help to CD8^+^ T cells and B cells, as well as modulating inflammatory and regulatory pathways. Previous studies have shown that eEF-2K influences CD4^+^ T cell metabolism and differentiation, particularly in the context of Th17 and regulatory T cell subsets. However, its role in the CD4^+^ T cell response to viral infections has not been fully elucidated. In the VACV infection model, it is plausible that eEF-2K deficiency in CD4^+^ T cells could modulate helper T cell activity, potentially influencing the quality and magnitude of the CD8^+^ T cell response indirectly. For example, enhanced metabolic activity and cytokine production by eEF-2K-deficient CD4^+^ T cells might further support antiviral CD8^+^ T cell responses or contribute to an altered inflammatory environment that enhances viral clearance. Future studies should investigate the phenotype, cytokine production, and helper function of CD4^+^ T cells in eEF-2K-deficient mice during VACV infection to provide a more comprehensive understanding of the immune landscape regulated by eEF-2K.

Taken together, this study provides novel insights into the role of eEF-2K in regulating CD8^+^ T cell responses during viral infection. Our results demonstrate that eEF-2K inhibits the generation and survival of VACV-specific effector CD8^+^ T cells via a TRAF3-mediated pathway. While our study highlights an increase in effector T cell numbers and survival in eEF2K⁻/⁻ conditions, the functional properties of these cells during the effector phase remain to be fully characterized. Future studies will incorporate functional assays to evaluate effector T cell responses comprehensively. Despite this limitation, our findings suggest that targeting eEF-2K can enhance the quantity of effector T cells without compromising their functionality or the formation of memory T cells. Thus, eEF-2K might be a promising therapeutic target for enhancing antiviral immunity. By leveraging the absence of eEF-2K to amplify T cell responses, future strategies could improve the efficacy of immune-based therapies against viral infections and potentially other diseases, such as cancer [29,30], where robust T cell responses are critical.

## Figures and Tables

**Table 1:** Antibody (Ab) usage.

Marker	Color	Company & Cat NO.
IL-2	BV421	BioLegend 503825
IFN-y	FITC	BioLegend 505806
CD8	PE	BioLegend 100708
CD44	FITC	BioLegend 103005
CD69	BV711	BioLegend 104537
CD197	BV421	BioLegend 120119
BCL-2	PE	Miltenyi Biotec 130-118-688
VACV tetramer	APC	NIH Tetramer core B8R
TRAF3	Alex Fluor 488	Proteintech CL488-66310

## Data Availability

The original contributions presented in this study are included in the article/supplementary material. Further inquiries can be directed to the corresponding authors.

## Supplementary Materials

The following supporting information can be downloaded at: https://www.mdpi.com/article/10.3390/v17010026/s1, Figure S1: eEF-2K does not alter the anti-apoptotic pathway in VACV-specific effector CD8⁺ T cells; Figure S2: Bar graphs highlighting key biological processes in the T cell response to viral infection.
