# Supplementary material for: eEF-2K Deficiency Boosts the Virus-Specific Effector CD8+ T Cell Responses During Viral Infection
Source: Viruses. 2024 Dec 28;17(1):26. doi: 10.3390/v17010026 (PMC11768472; doi:10.3390/v17010026)
Supplement: Supplementary file 1 [file viruses-17-00026-s001.zip › viruses-3388432-supplementary.pdf]

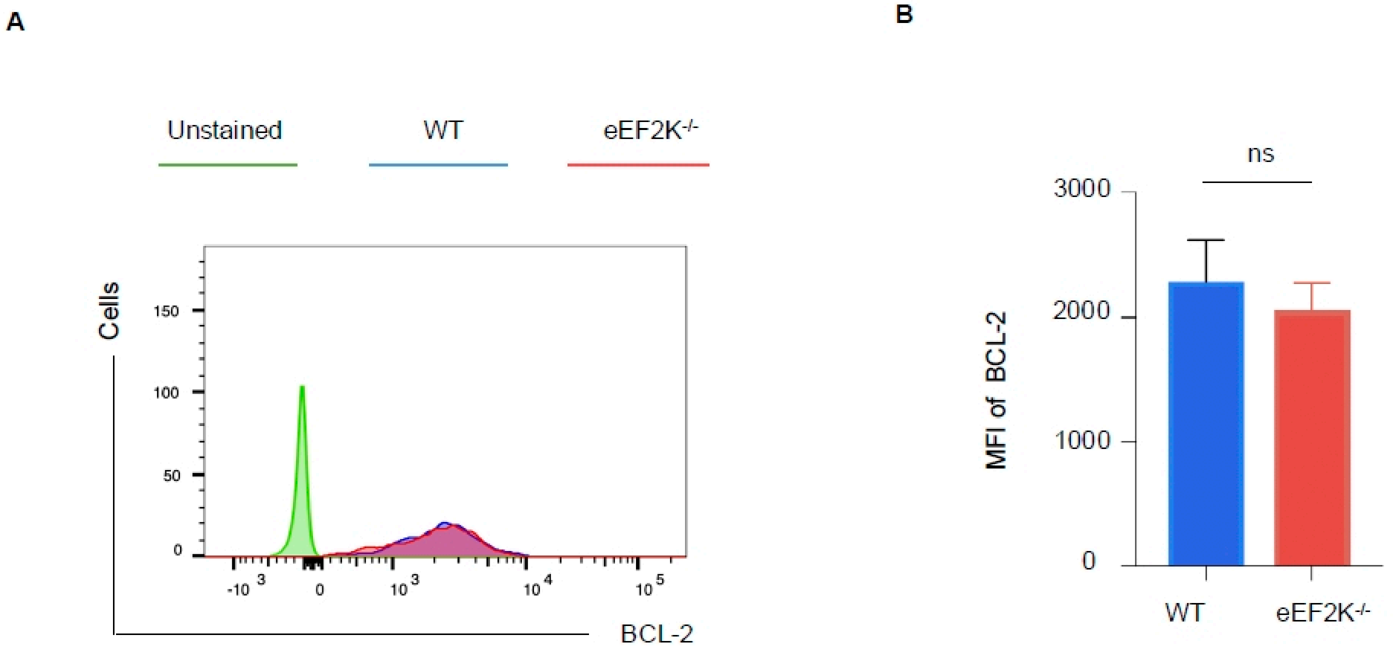

Figure S1. eEF-2K does not alter the anti-apoptotic pathway in VACV-specific effector CD8⁺ T cells. Expression of the anti-apoptotic protein BCL-2 was assessed in CD8⁺B8R⁺ T cells from WT and eEF-2K$^{-/-}$ mice post-VACV infection. (**A**) Representative flow cytometry plots of intracellular BCL-2 expression. (**B**) MFI quantification of BCL-2 expression in CD8⁺B8R⁺ T cells.

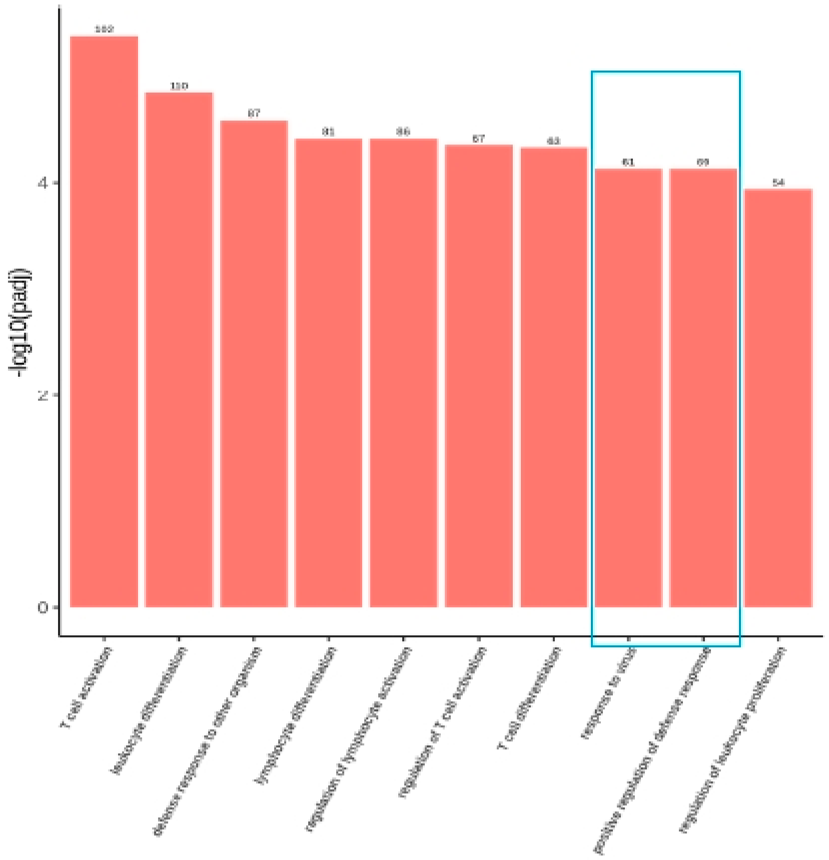

Figure S2. Bar graphs highlighting key biological processes in the T cell response to viral infection. Bar graphs represent biological processes related to the T cell response to viral infection and positive regulation of immune defense, based on GO analysis of differentially expressed genes.
